# Mouse Dendritic Cells Pulsed with Capsular Polysaccharide Induce Resistance to Lethal Pneumococcal Challenge: Roles of T Cells and B Cells

**DOI:** 10.1371/journal.pone.0039193

**Published:** 2012-06-18

**Authors:** Noam Cohen, Raanan Margalit, Meirav Pevsner-Fischer, Simon Yona, Steffen Jung, Lea Eisenbach, Irun R. Cohen

**Affiliations:** Department of Immunology, the Weizmann Institute of Science, Rehovot, Israel; Albany Medical College, United States of America

## Abstract

Mice are exceedingly sensitive to intra-peritoneal (IP) challenge with some virulent pneumococci (LD50 = 1 bacterium). To investigate how peripheral contact with bacterial capsular polysaccharide (PS) antigen can induce resistance, we pulsed bone marrow dendritic cells (BMDC) of C57BL/6 mice with type 4 or type 3 PS, injected the BMDC intra-foot pad (IFP) and challenged the mice IP with supra-lethal doses of pneumococci. We examined the responses of T cells and B cells in the draining popliteal lymph node and measured the effects on the bacteria in the peritoneum and blood. We now report that: 1) The PS co-localized with MHC molecules on the BMDC surface; 2) PS-specific T and B cell proliferation and IFNγ secretion was detected in the draining popliteal lymph nodes on day 4; 3) Type-specific resistance to lethal IP challenge was manifested only after day 5; 4) Type-specific IgM and IgG antibodies were detected in the sera of only some of the mice, but B cells were essential for resistance; 5) Control mice vaccinated with a single injection of soluble PS did not develop a response in the draining popliteal lymph node and were not protected; 6) Mice injected with unpulsed BMDC also did not resist challenge: In unprotected mice, pneumococci entered the blood shortly after IP inoculation and multiplied exponentially in both blood and peritoneum killing the mice within 20 hours. Mice vaccinated with PS-pulsed BMDC trapped the bacteria in the peritoneum. The trapped bacteria proliferated exponentially IP, but died suddenly at 18–20 hours. Thus, a single injection of PS antigen associated with intact BMDC is a more effective vaccine than the soluble PS alone. This model system provides a platform for studying novel aspects of PS-targeted vaccination.

## Introduction

The protective immune response to a potentially pathogenic agent is a complex phenomenon involving activations of innate and adaptive immune cells in response to target antigens, the elaboration of effector mechanisms, and effects on the pathogen – all progressing at specific times and in different anatomic compartments in the host body. Here we set out to develop a comprehensive model system that could serve as a platform for observing different aspects of the immune response and for detecting particular immune reactions in need of in-depth analysis. We focused on inducing resistance in mice to IP challenge with highly virulent *Streptococcus pneumoniae* – a pathogen of which one bacterium suffices to kill 50–100% of naïve mice within 24 hours. The target antigen was the capsular polysaccharide (PS).

The pathogenicity of *Streptococcus pneumoniae* has been attributed to the PS antigen of the bacterial surface [1], [2]. There are about 90 different pneumococcal PS serotypes that act as the major virulence factor of the bacteria. Vaccines against pneumococci have been traditionally based on PS antigens, and anti-PS antibodies have been known to mediate resistance to the bacterial infection [3], [4]. However, PS vaccines are poorly immunogenic, especially in young children, the elderly, and immunosuppressed persons [5], [6], [7]. The PS antigens are T-cell independent type 2 (TI-2) and activate B cells directly to secrete IgM Abs with no immunological memory. A new generation of pneumococcal vaccines has been designed in which the PS antigen is conjugated to a carrier protein immunogenic for helper T cells [8]. However, there have been few studies of the possible role of innate mononuclear antigen presenting cells like dendritic cells (DC) and macrophages in the activation of the immune response to the PS.

DC are professional APC, able to internalize exogenous antigens, migrate to draining lymph nodes (LN) and prime T cells [9], [10], [11]. These activities are enhanced by inflammatory components that stimulate toll-like receptors (TLR) inducing DC maturation. Previously we showed that TLR4 stimulation of macrophages or bone marrow-derived dendritic cells (BMDC) in vitro followed by pulsing with pneumococcal PS type 4 (PS4) led to the internalization of the PS followed by its appearance on the cell surface for prolonged times and, upon IP injection to naïve mice, induced long-lasting, type-specific resistance to challenge IP with lethal numbers of pneumococci. This resistance could not be accomplished by immunization with soluble PS4 [12].

In the present study we investigated factors important for successful pneumococcal vaccination by PS-pulsed BMDC, including the nature of the host immune response and the mechanism of resistance. We injected mice intra-footpad (IFP) with soluble PS, with unpulsed BMDC, or with the PS-pulsed BMDC and challenged the mice intraperitoneally (IP) with lethal doses of pneumococci. We investigated the cellular responses developing in the draining popliteal LN and the effects exerted on the bacteria as a function of induced resistance.

## Results

### Immunization with PS4-BMDC either IP or IFP induces resistance to Pn4 bacteria injected IP

**Figure 1:**
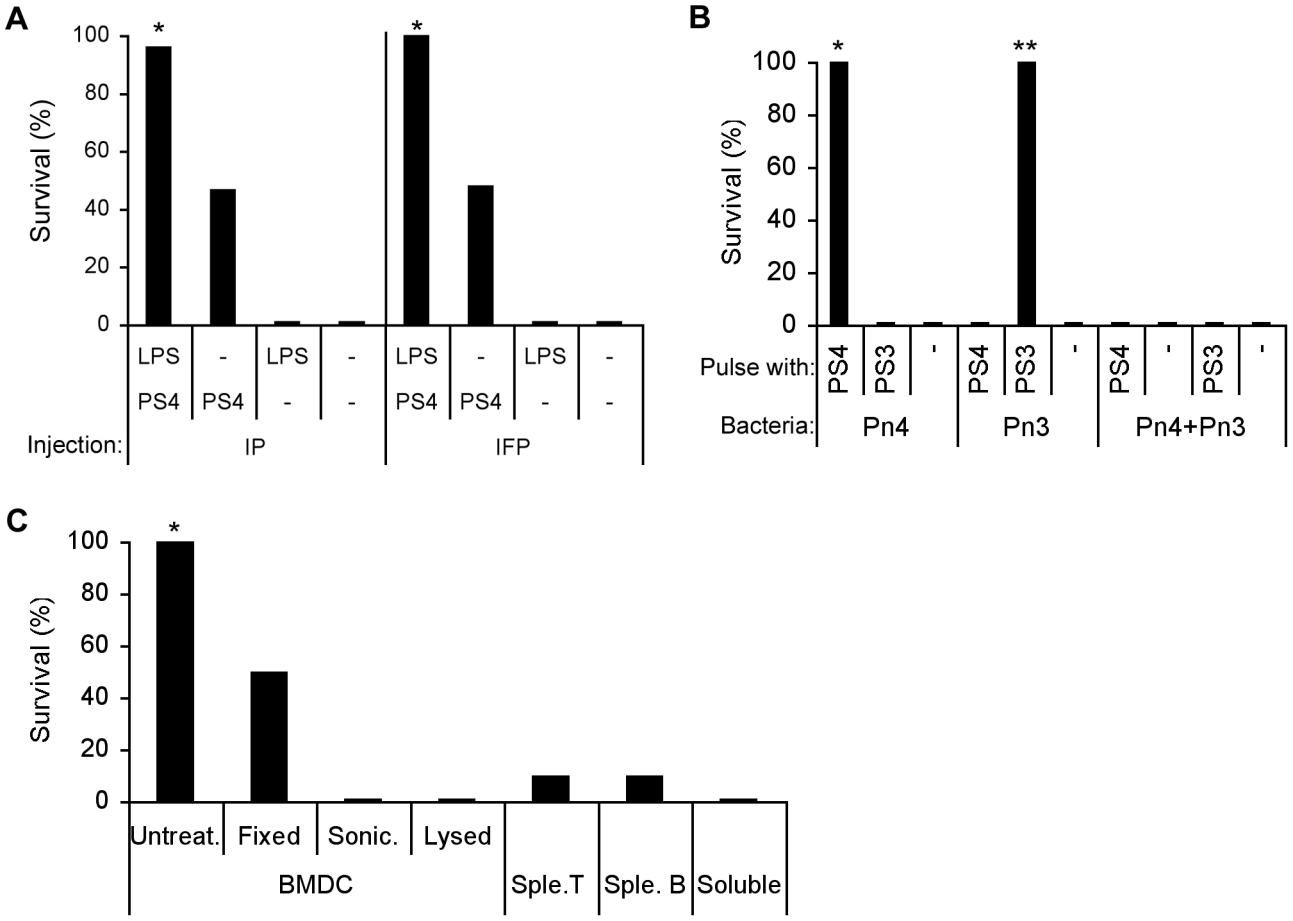
Vaccination with type-specific PS-pulsed BMDC induces type-specific resistance: Only intact BMDC are effective. (A) LPS-stimulated (LPS) and non-stimulated (−) BMDC were pulsed with PS4 and injected once IP or IFP to C57BL/6 mice (5×10^6^ cells per mouse). Three-weeks later, the mice were challenged with Pn4 (100,000×LD_50_ CFU) IP: *p<0.05 +LPS+PS4 compared to −LPS+PS4, +LPS−PS4 and −LPS−PS4, n = 25. (B) LPS-stimulated or non-stimulated BMDC were pulsed with PS4 or PS3 and injected IFP to C57BL/6 mice. Three weeks later, the mice were challenged with Pn4 or Pn3 or with a mixture of both Pn4 and Pn3 IP: *p<0.05 +LPS+PS4, Pn4 compared to +LPS+PS3, Pn4, +LPS−PS, Pn4 and to all groups of Pn4+Pn3. **p<0.05 +LPS+PS3, Pn3 compared to +LPS+PS4, Pn3, −LPS−PS, Pn3 and to all groups of Pn4+Pn3, n = 15. (C) LPS-stimulated, PS4-pulsed BMDC were fixed with glutaraldehyde 1%, lysed with DDW, sonicated (sonic.) or were not treated (untreat.). The cells were then injected into recipient mice. Control LPS-stimulated and PS4-pulsed splenic (sple.) T and B cells or soluble LPS+PS4 were injected to other groups of mice. After three weeks, the mice were challenged with Pn4 (100,000×LD_50_): *p<0.05 untreated BMDC compared to all groups, n = 10.

We previously reported that resistance to a lethal challenge with Pn could be induced by vaccination with a macrophage line or with BMDC that had been pulsed with the PS of the challenge Pn serotype. Moreover, macrophages or BMDC that had been activated with a TLR4 agonist were more effective vaccines [12]. In these experiments, both the vaccination and the challenge were carried out IP, and one could argue that the resistance to challenge was induced locally rather than systemically. To examine this issue, we vaccinated mice with PS4-pulsed BMDC administrated either IP or IFP (5×10^6^ cells per mouse); the BMDC had been activated or not with the TLR4 ligand LPS before being pulsed with PS4. Three weeks later, the mice were challenged IP with a supra-lethal dose of Pn4 (LD_50_×100,000). Figure 1A shows that both IFP and IP administration were equally effective in inducing resistance to the IP challenge. As we reported earlier [12], LPS-activated BMDC were more effective vaccines. Thus, vaccination with PS4-pulsed BMDC IFP was as effective as IP vaccination. In the experiments reported here, we vaccinated IFP and challenged IP.

### Vaccination is PS-specific and does not induce non-specific bystander protection

To examine whether vaccination with PS-pulsed BMDC is serotype specific, we vaccinated mice with BMDC pulsed with either PS type 4 or PS type 3, two different serotypes of pneumococcal PS with different carbohydrate structures [13], [14]. Three weeks later, the mice were challenged IP with Pn bacteria type 4 or type 3 or with a mixture of Pn4 and Pn3. Figure 1B shows that protection was PS type-specific: mice immunized with PS4 were protected against Pn4 and not against Pn3 and vice versa. In addition, mice immunized with PS4-BMDC or with PS3-BMDC were not protected against a challenge with both Pn3 and Pn4 mixed together injected IP; this indicates that the effector mechanism that kills the Pn is antigen specific and does not affect adjacent bacteria. The following experiments were done to explore the requirements for the induction of resistance.

### Intact PS4-pulsed BMDC are required to induce resistance

Previously we reported that intact RAW macrophage line cells were required to vaccinate recipient mice: Living irradiated RAW or intact RAW cells killed by fixation were effective, but lysed RAW did not induce resistance [12]. To see if BMDC behaved similarly, we treated PS4-pulsed BMDC in three ways before vaccination: lysis of the living cells; sonication of the living cells; or glutaraldehyde (GH)-fixation of the cells. Figure 1C shows that the GH-fixed BMDC vaccine was less effective than live, intact BMDC. Lysed or sonicated BMDC or the injection of soluble LPS and PS4 were not effective at all in inducing resistance. Also ineffective was the injection of the same number of T cells or B cells that had been incubated with LPS and pulsed with PS4. Therefore the induction of resistance to Pn challenge is a property of intact, live BMDC that have been activated by LPS and pulsed with PS4. Intact BMDC fixed with GH were partially effective vaccines.

### Pulsed PS appears on the surface of both LPS-stimulated and non-stimulated BMDC in association with MHCI, II and CD1d presenting molecules

**Figure 2:**
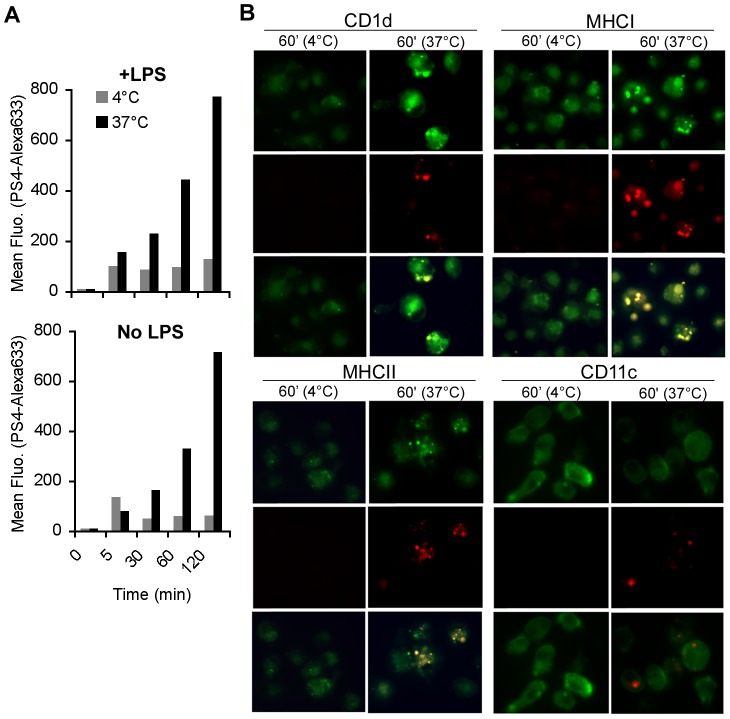
PS4 is present on the surface of BMDC in association with presenting molecules. BMDC were stimulated with LPS and pulsed with Alexa633-labeled PS4 for 5, 30, 60 and 120 minutes at 37°c or 4°c. The cells were then washed and monitored by flow cytometry (A), or were also stained for surface markers: anti-CD1d-PE, anti-H-2K^b^(MHCI)-FITC, anti-I-A^b^(MHCII)-PE, CD11c-PE, and were examined by fluorescent microscopy (B). Surface marker staining is presented in green; Alexa633-PS4 in red; the co-localization of both of them is detected by the yellow color.

Previously we reported that LPS stimulation of RAW cells induced the late appearance of PS4 on the cell surface in clusters [12]. Here, we examined the presence of pulsed PS4 on the surface of LPS-stimulated or non-stimulated BMDC, and tested whether the PS4 is associated with presenting molecules on the cell surface. LPS-stimulated or non-stimulated BMDC were pulsed with PS4 that had been labeled with Alexa633 and the presence of labeled PS4 was assayed by flow cytometry and by fluorescent microscopy at various times after incubation at 37°c or on ice. Labeled PS4 appeared equally on the surface of LPS-stimulated and non-stimulated BMDC in increasing amounts over 2 hours (Fig. 2A), but only when the BMDC were incubated at 37°c. PS4 was present on the surface in clusters after 60 minutes incubation at 37°c and in co-localization with CD1, MHC-I and MHC-II presenting molecules, but not in association with the non-presenting molecule CD11c (Fig. 2B). The level of PS4 associated with the BMDC rose with time at 37°c but the PS4 level remained low when the BMDC were incubated on ice (Fig. 2A); this indicates that surface presentation of PS4 is a process that requires a physiological temperature. In contrast to what was found in macrophages [12], BMDC showed the same level of PS4 on the surface whether the cells had been stimulated or not with LPS, although the LPS-stimulated BMDC were more effective than the non-stimulated BMDC in the induction of resistance to the bacterial challenge (Fig. 1A).

### Presenting, co-stimulatory and migrating molecules expressed on PS4-BMDC are important but not necessary to induce resistance

**Figure 3:**
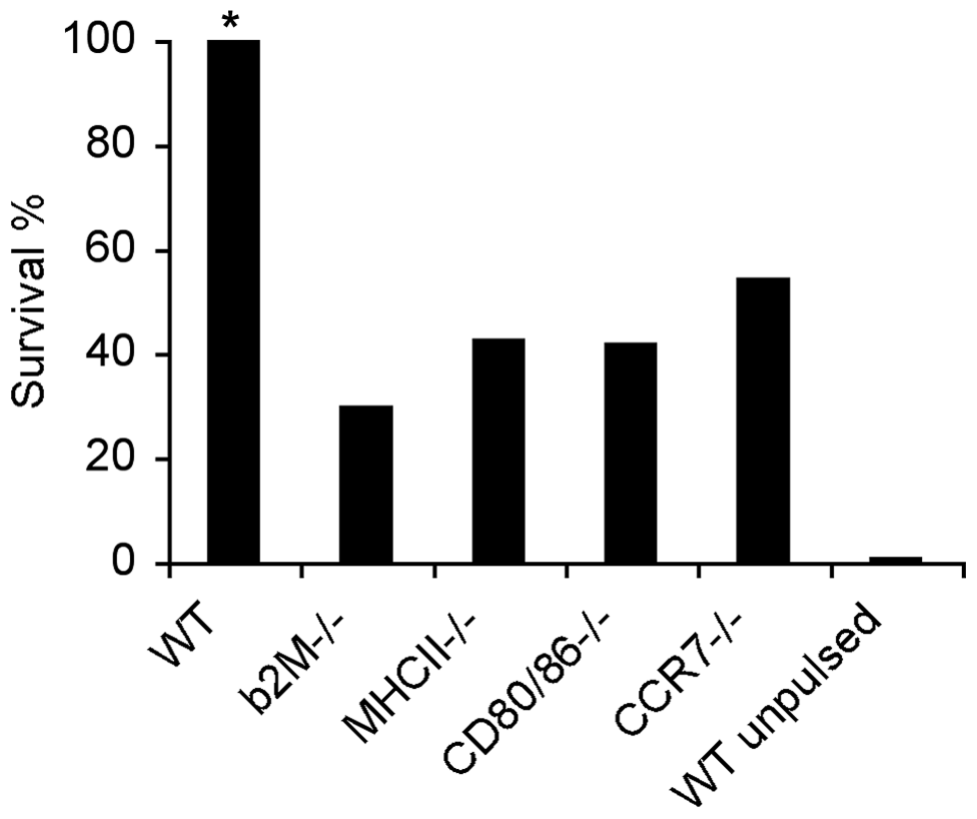
Deficiency of presenting and migrating molecules expressed on PS4-BMDC reduces the efficacy of induced resistance. BMDC derived from β2M, MHCII, CD80/86, CCR7 deficient or WT C57BL/6 mice were stimulated with LPS, pulsed with PS4 and injected IFP into WT mice. Un-pulsed WT BMDC were injected as a control (WT un-pulsed). Three weeks later, the mice were challenged with Pn4 IP. *p<0.05 WT compared to all the groups, n = 15–20.

We showed above that the pulsed PS4 appears on the surface of the BMDC clustered with molecules known to be involved in antigen presentation (Fig. 2B). To learn whether expression of these presenting molecules on the injected BMDC is required for the induced resistance, we used BMDC derived from mice genetically lacking beta2M (MHCI and CD1 deficient) or lacking MHC class II molecules. In addition, we examined the effectiveness of BMDC that lacked the expression of CD80 and CD86, costimulatory molecules required for T cell priming, and of CCR7, a chemokine receptor molecule required for DC migration [15]. The BMDC derived from these C57BL/6 deficient mice and from control wild-type mice were pulsed with PS4 and injected into wild-type C57BL/6 mice. Three weeks later the mice were challenged with a supra-lethal dose of Pn. Figure 3 shows that the vaccinations with PS4-BMDC that lacked the presenting, co-stimulatory or migrating molecules were about 50% less effective compared to vaccination with wild-type BMDC. Nevertheless, a significant number of mice vaccinated with these deficient BMDC did resist challenge compared to non-vaccinated control mice. These results suggest that the association of PS4 on the surface with MHC class I and class II presenting molecules and the expression of CD80, CD86 and CCR7 molecules is important for the induction of the most effective immune resistance to Pn4 challenge. However, a significant measure of resistance can be induced in the absence of each of these molecules alone on the pulsed BMDC. This finding raises the question of whether the PS antigen might be taken up by antigen-presenting cells resident in the host mice.

### PS4 on the surface of the injected BMDC is transferred to host cells

**Figure 4:**
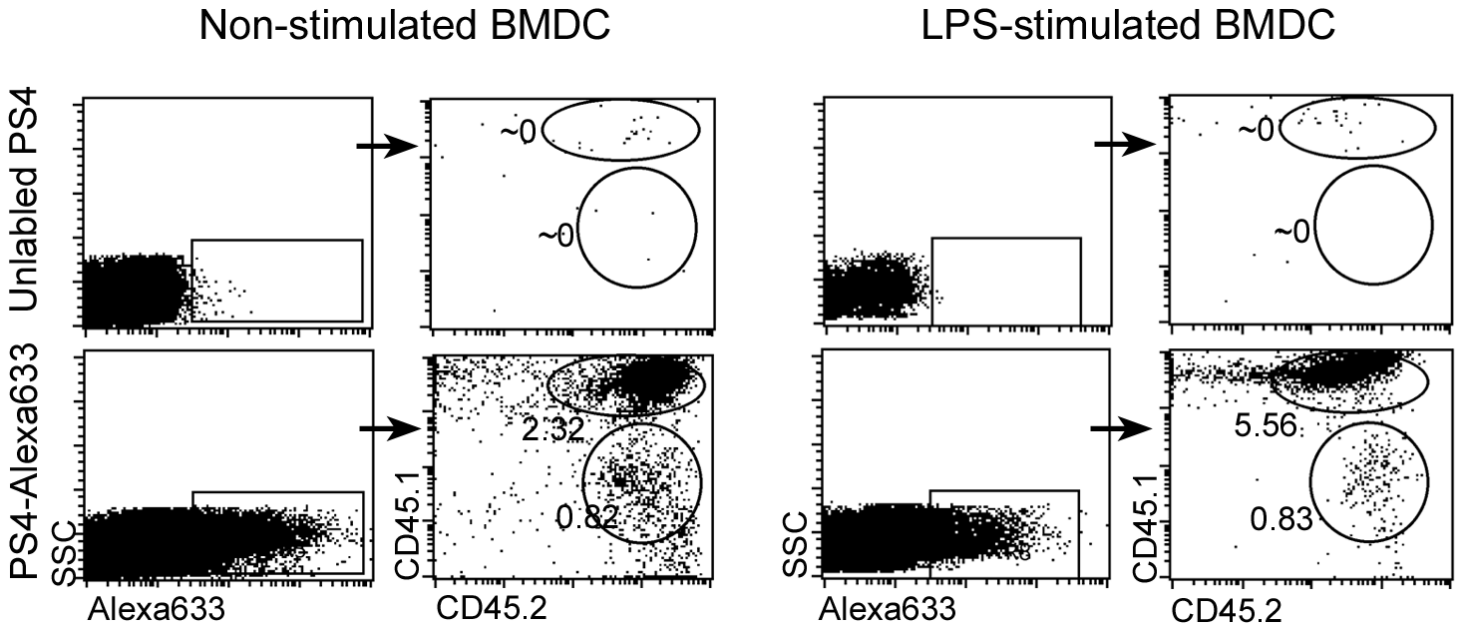
PS4 on the surface of BMSC is transferred to endogenous cells in the peritoneum. BMDC derived from CD45.2 mice (donors) were stimulated with LPS and pulsed with PS4-Alexa633. The cells were injected IP into CD45.1 mice (recipients). After 24 hours, the mice were sacrificed, cold PBS was injected IP and the peritoneal fluid was collected. The collected cells were stained with anti-CD45.1-PE and anti-CD45.2-FITC, and were analyzed by flow cytometry. The numbers indicate the percentage of labeled cells.

To detect the possible transfer of PS4 from the injected donor BMDC to host cells, we injected the donor cells IP and tested whether labeled PS4 could be detected in host cells isolated from the peritoneum. For these experiments we used the allotypic mouse system: Donor BMDC derived from CD45.2 mice were stimulated with LPS for 24 hours and pulsed with PS4-Alexa 633 for 1 hour. The cells were then injected IP to CD45.1 host mice. As a control, BMDC pulsed with unlabelled PS4 were also injected. After 24 hours, the mice were sacrificed and the peritoneal fluid was collected. The peritoneal cells were analyzed by FACS to detect the labeled PS4. PS4 was found in association with both CD45.2 donor BMDC and CD45.1 host cells (Fig. 4). In fact, more CD45.1 host cells were co-labeled with PS4-Alexa633 compared to the CD45.2 donor cells. These results suggest that the induction of the immune response to the PS4 involves both the PS4 present on the surface of the injected BMDC and the transfer of PS4 to host cells, possibly antigen presenting cells.

It is important to note, however, that injection of the PS4 antigen alone or of lysed PS4-BMDC failed to induce resistance to challenge (Fig. 1C); under these circumstances, PS4 was not rendered immunogenic by host APC. Thus, intact antigen-pulsed donor BMDC are required to realize the possible contribution of host monocytes or other cells to the resistance to lethal challenge measured in these studies. The following series of experiments were done to gain some insights into the immune response of the hosts responsible for the resistance induced by the donor PS4-BMDC.

### Immunization IFP induces lymphocyte proliferation in the draining popliteal LN on day 4

**Figure 5:**
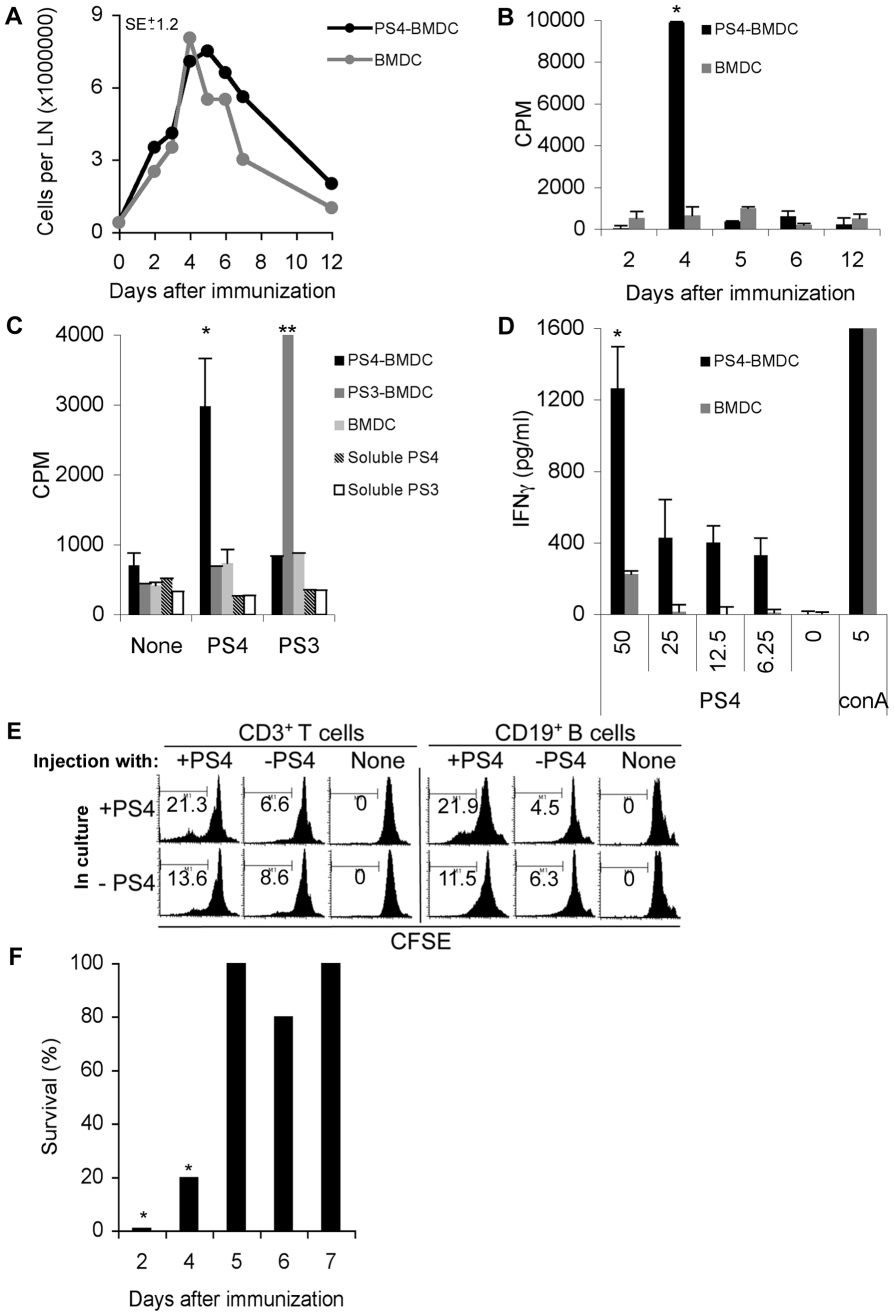
Immunization IFP induces proliferation of draining popliteal lymphocytes 4 days later; systemic resistance appears 5 days later. (A) Mice were immunized IFP with LPS-stimulated, PS4-pulsed BMDC (PS4-BMDC) or with LPS-stimulated, un-pulsed BMDC (BMDC). Two, three, four, five, six, seven and twelve days later, the draining popliteal LN were isolated, pooled, and mashed and cell counts were performed to determine the total numbers of cells in the LN. The standard error (SE) is +/−1.2, p>0.05. (B) The isolated, pooled lymphocytes of each group were incubated with PS4 for 85 hours supplemented with H^3^-labeled thymidin (added in the last 12 hours). *p<0.05 LPS+DC+PS4, 4 hours compared to all. (C) Mice were immunized IFP with PS3-BMDC, PS4-BMDC, un-pulsed BMDC, LPS+PS3 or LPS+PS4. Four days after immunization, draining popliteal LN were isolated, mashed, and incubated in 96-well plates with PS3 or PS4 and H^3^-labeled thymidine. *p<0.05 compared to all PS4 and none groups. **p<0.05 compare to all PS3 and none groups. (D) Mice were immunized IFP with PS4-BMDC or with un-pulsed BMDC. Four days after immunization, draining popliteal LN were isolated, incubated with PS4 in different doses (0–50 µg/ml) or with Concanavalin A (conA) (1.25 µg/ml), a strong mitogen, for 85 hours. The supernatants were collected and examined for the presence of IFNγ by ELISA. *p<0.05 lymphocytes of PS4-BMDC injected mice compare to all. (E) Isolated lymphocytes were stained with CFSE and incubated with PS3 or PS4 for 85 hours. The cells were then collected, stained for anti-CD3-biotin: SA-cy5 or anti-CD19-APC Ab and followed by flow cytometry. (F) Mice were immunized with PS4-BMDC IFP. Two, four, five, six and seven days later, the mice were challenged with Pn4 bacteria (100,000×LD_50_) IP. *p<0.05 two or four days after immunization compare to all groups.

The first lymphoid organ that might be expected to respond to activated BMDC introduced into the subcutaneous tissue of the foot pad would be the draining popliteal LN. To examine the kinetics of lymphocyte activation in the draining LN, we conducted a time-course experiment: mice were immunized IFP with BMDC unpulsed or pulsed with PS4, and after 2, 4, 5, 6 or 12 days, the mice were sacrificed and the popliteal draining LN cells were isolated. Figure 5A shows that the numbers of LN cells increased from ∼1×10^6^ cells in the naïve mice (time 0) to ∼8×10^6^ cells after 4 days, then decreased to ∼2×10^6^ cells after 12 days. There was no significant difference in the numbers of LN cells in the PS4-BMDC and BMDC injected mice. Yet the PS4-BMDC injected mice were resistant to the Pn4 challenge whereas the BMDC injected mice were not (Fig. 1A). The LN cells of the mice that had been injected with PS4-BMDC also manifested a proliferative response to PS4: Figure 5B shows that proliferation of lymphocytes was detected only on day 4 after IFP injection of PS4-BMDC, but not before or after. Mice that had been injected IFP with unpulsed BMDC showed no proliferative response to PS4. Moreover, the immunization IFP with PS4-BMDC did not induce activation of the non-draining LN or spleen at any of the time points measured (data not shown).

To examine whether the proliferation was antigen-specific, we immunized mice with BMDC pulsed with either PS4 or PS3, or with soluble PS4 or PS3+LPS and examined the proliferation of cells from the draining popliteal LN 4 days later. Figure 5C shows that the LN cells of the PS4-BMDC injected mice proliferated specifically to PS4 and not to PS3, whereas LN cells of the PS3-BMDC injected mice responded to PS3 and not to PS4. Cells isolated from the mice that had been injected with soluble LPS+PS4 or LPS+PS3 did not proliferate (Fig. 5C) and were similar in size to naïve un-injected mice (data not shown).

### IFNγ secretion by LN cells

Proliferation of lymphocytes is often associated with the secretion of cytokines. The supernatants of the cultured LN cells were collected and tested by ELISA for IFNγ (for a Th-1 type response), IL-17 (for a Th-17 type response) or IL-10 (for a Th-2 type response). Figure 5D shows that the proliferating lymphocytes secreted IFNγ specifically when cultured with PS4, in a dose-dependent manner. No IL-17 or IL-10 was detected in the supernatants, suggesting a Th-1 type response.

Thus induction of resistance to bacterial challenge (Figure 1A) is associated with serotype-specific proliferation and cytokine secretion detected in the draining LN; soluble PS failed to induce either proliferation or resistance.

### Both B cells and T cells proliferate on day 4 in the popliteal LN

To examine which population of LN cells proliferated, we isolated cell suspensions from the draining LN 4 days after IFP injection of PS4-BMDC, labeled the LN cells with the fluorescent dye CFSE and cultured the cells with or without PS4. After 85 hours in culture, the cells were collected, stained with anti-CD3 Abs (to detect T cells) or anti-CD19 Abs (to detect B cells) and assayed by flow cytometry. Figure 5E shows that immunization IFP induced PS4-specific proliferation of both CD3+ T cells and CD19+ B cells. Popliteal LN of mice immunized with un-pulsed BMDC did not manifest cell proliferation.

### Resistance to Pn4 challenge is manifested only after 5 days

Since lymphocyte proliferation was detected in the draining LN only after 4 days, we investigated whether the minimal time required for the induction of resistance might be associated with this antigen-specific T cell and B cell proliferation. Mice were immunized with PS4-BMDC and then were challenged IP after 2, 4, 5, 6, or 7 days. Resistance to challenge IP was observed only after 5 days (Fig. 5F), suggesting that the minimal time required for the processes necessary for the establishment of resistance in the peritoneum is subsequent to antigen-specific lymphocyte proliferation in the draining LN. The following experiments were done to learn whether induced resistance depended on recipient T cells, B cells or both.

### Immunized mice deficient of B cells are susceptible to the Pn4 challenge, whereas, immunized mice deficient of αβT cells or γδT cells acquire resistance

**Figure 6:**
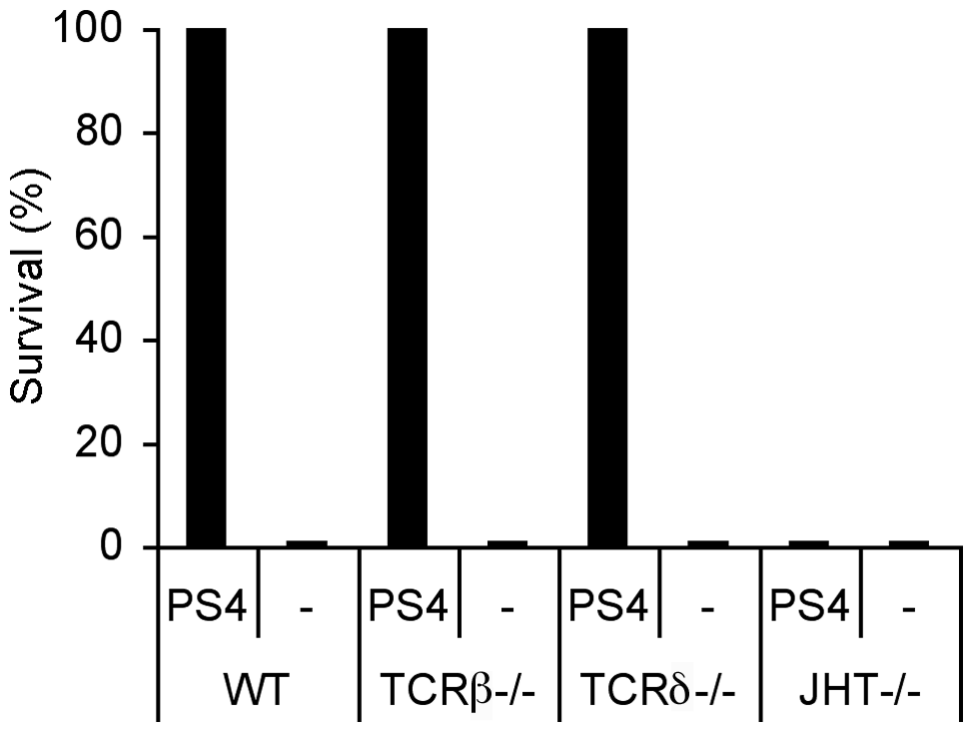
Mice deficient of B cells (JHT−/−) immunized with PS4-BMDC are susceptible to Pn4 challenge. WT, B-cell deficient (JHT−/−), αβT cell deficient (TCRβ−/−) or γδT cell deficient (TCRδ−/−) mice were immunized IFP with PS4-BMDC. Three weeks later, the mice were challenged with Pn4 bacteria (100,000×LD_50_) IP (n = 10).

It has been reported that a rapid immune response to bacterial infection is induced by γδT cells that respond within hours [16]. IFNγ secretion by T cells can originate from Th-1 helper T cells or from γδT cells. To test whether the induction of resistance requires functional T cells (conventional αβT or γδT cells) or B cells, we immunized mice deficient of conventional T cells (TCRβ−/−), γδT cells (TCRδ−/−) or B cells (JHT) with PS4-BMDC. Three weeks later, we tested the resistance of the mice to Pn challenge. Surprisingly, mice deficient in γδT cells or αβT cells were resistant whereas mice deficient in B cells were susceptible to the Pn4 challenge (Fig. 6). This result indicates that the recipient B cells are required for the induced resistance; αβT cells or γδT cells appear not to be essential in the mechanism of the induced resistance, although proliferation of PS-specific CD3+ T cells and IFNγ secretion were observed in association with the proliferation of B cells and the acquisition of resistance in mice with intact T cell populations (Fig. 5D).

### Immunization with PS-pulsed BMDC induces specific anti-PS IgM and IgG Ab production, however, Abs could not be detected in the sera of some of the protected mice

**Figure 7:**
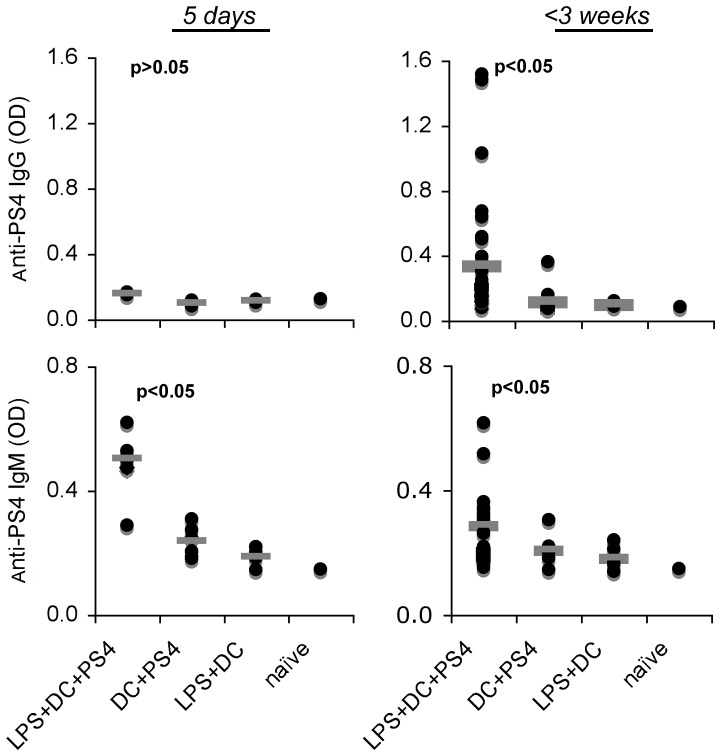
Immunization with PS4-BMDC induces short-term anti-PS4 IgM and long-term anti-PS4 IgG Ab. Mice were immunized with PS4-BMDC either IP or IFP. Five days and three weeks after immunization, the mice were bled, and serum samples were tested for the presence of anti-PS4 IgM and IgG Abs by ELISA. p<0.05 tested by Wilcoxon non-parametric test (n = 10–25).

We assayed the serum levels of anti-PS4 IgM and IgG developing in mice that had been immunized IFP with PS4-pulsed BMDC, activated or not with LPS, or with LPS-activated BMDC. The mice were bled after 5 days or 3 weeks, and serum samples were tested for anti-PS4 Ig Abs by ELISA. Figure 7 shows that all the mice immunized with PS4-pulsed, LPS-activated BMDC manifested anti-PS4 IgM Abs 5 days after immunization that significantly declined by three weeks; these Abs were not detected in the sera of mice that had been injected with un-pulsed BMDC or with pulsed BMDC that had not been activated with LPS. However, only about 20% of the mice that had been injected with pulsed, LPS-activated BMDC developed detectable anti-PS4 IgG Ab at 3 weeks, although all these mice were resistant to the Pn4 challenge. Moreover, bacterial challenge did not induce detectable IgG antibodies in mice that had resisted the challenge (in three independent experiments, data not shown). Notably, no anti-PS4 IgE and IgA Abs were detected (data not shown).

It was conceivable that sera that tested negative in the ELISA assay might still contain functionally protective antibodies. We performed passive serum transfer experiments in an attempt to demonstrate such antibodies. Passive protection from bacterial challenge (100×LD_50_) could be demonstrated by passive transfer of 0.2 ml of serum from donors with antibody-positive sera, but 2 ml of sera from the IgG antibody-negative mice failed to protect (data not shown). These results collectively show that immunization with BMDC pulsed with PS4 indeed induces a PS4-specific B cell response that is essential for acquired resistance; however the resistance is not necessarily associated with the presence of protective anti-PS4 Abs detectable in the serum.

### Following Pn challenge in vaccinated mice, the bacteria are sequestered in the peritoneum and die after initial replication

**Figure 8:**
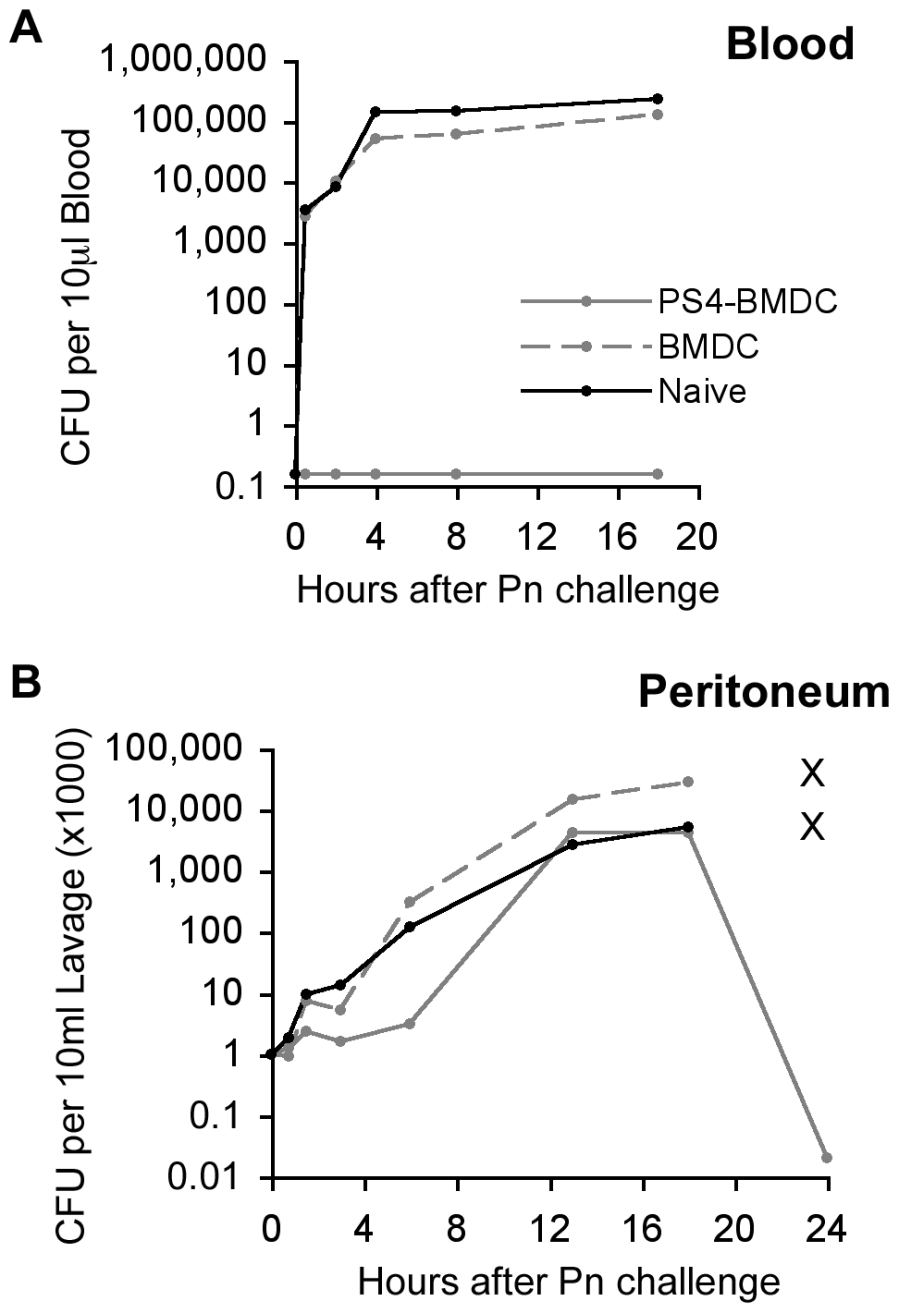
Challenge bacteria are trapped in the peritoneum of vaccinated mice and do not enter the blood or kill the mice; the vaccinated mice kill the bacteria after 18–24 hours. Mice were immunized IFP with PS4-BMDC or with un-pulsed BMDC. Three weeks later, the mice were challenged with Pn4 bacteria (100,000×LD_50_) IP. The mice were bled 0.5, 2, 4, 8, 18 and 24 hours post-challenge, and peritoneal fluids were collected. The peritoneal fluid (A) and the blood samples (B) were plated on agar overnight and CFU were then counted. Note: x means that the mice died and the samples could not be collected.

The final experiments were done to gain some insight into the fate of the challenge bacteria in the peritoneum and blood of vaccinated and control mice. Mice were immunized or not with PS4-BMDC, and, three weeks later, the mice were challenged with the Pn4 bacteria (100,000×LD_50_) and were bled and then sacrificed at different time points. Sera and peritoneal lavage samples were examined for numbers of live bacteria by the CFU counting assay. Figure 8A shows that not a single bacterium could be detected 0.5–18 hours after Pn challenge in the blood of mice that had been immunized with PS4-BMDC. In contrast, the mice immunized with un-pulsed-BMDC, like the naïve mice, manifested dissemination of the bacteria to the blood and their logarithmic replication over 4 hours. The numbers of bacteria reached a plateau and all the control vaccinated mice died by 20 hours after challenge.

The fate of the bacteria in the peritoneum was surprising: Although not a single bacterium could be detected in the blood of the PS4-BMDC immunized mice and all the mice resisted challenge, the injected Pn4 bacteria replicated in the peritoneum at the same logarithmic rate as did the bacteria in the control vaccinated or naïve mice for about 18 hours, after that time point, all the bacteria died in the effectively vaccinated mice. The naïve and control-vaccinated mice died with great numbers of bacteria in both peritoneum and blood (Fig. 8B). Thus the protective mechanism induced by PS4-BMDC vaccination upon challenge immediately confines the bacteria to the site of injection but does not prevent their replication; an immune response effector mechanism kills them only about 18 hours later.

## Discussion

The experiments described here were undertaken to obtain a comprehensive look at the induction of resistance to lethal Pn challenge induced in mice by intra footpad injection of PS-pulsed BMDC. The PS-pulsed BMDC vaccine was highly effective in inducing PS-specific resistance to a supra-lethal pneumococcus challenge; immunization with a PS serotype 4 did not induce resistance to Pn type 3 bacteria, and *vice versa*. The PS-pulsed BMDC vaccine induced long-term systemic protection; and immunization IFP conferred protection for more than three months to the bacterial challenge IP. In contrast, immunization with a single dose of soluble PS (100 µg/ml), with or without the addition of LPS, did not induce resistance. Moreover, the resistance appeared to require an intact BMDC cell surface; lysis or sonication of PS4-pulsed BMDC abolished the induction of protection, whereas fixation reduced the efficacy of PS4-pulsed BMDC by 50%. These findings collectively emphasize the requirement of intact PS4-pulsed BMDC to induce resistance. Injection of PS4-pulsed BMDC IP led to the transfer of PS4 to endogenous host cells. However, a single injection of lysed PS4-BMDC or of PS4 antigen alone failed to induce resistance to the challenge. Although we could not measure the exact amount of PS4 released from the lysed BMDC, it would seem that soluble PS4 at doses of 2.5 µg IFP or 20 µg IP were not rendered immunogenic by host APC.

PS on the intact surface of BMDC was visually associated with presenting molecules MHCI, MHCII and CD1d (Fig. 6B). Indeed, BMDC lacking these presenting or migrating molecules (CD/80, CD/86 and CCR7) were significantly less effective in inducing resistance. Presentation of PS molecules is not widely reported in the literature. However there are some indications that support this finding: a zwitterionic PS (having both positive and negative charges) of *B. fragilis* was shown to be presented to T cells via the canonical MHC class II pathway [17], [18]. Pneumococcal PS molecules were shown to be taken-up by immature human DC and transported to HLA-DR and late endosomal compartments [19]. The lipid determinants of glycolipid antigens were shown to activate specific T cells *via* presentation on CD1 [20]. Lai et al reported that the glycoconjugate is processed and the PS is associated with MHCII on the cell surface of APC [21]. Further studies are needed to investigate the molecular interaction of PS4 (a non-zwitterionic molecule) with MHC molecules; it is not clear whether the clusters of the PS4 we observed on the BMDC surface form an immunological synapse.

Most anti-bacterial vaccines are evaluated by their ability to induce high titers of IgG Ab to relevant epitopes. Specifically, the requirement of anti-PS Ab for the clearance of Pn infection has long been reported [22], [23], [24]. Indeed, we showed that B cells are necessary for the induction of resistance and that the resistance to challenge by PS-pulsed BMDC was associated with the appearance of specific Abs in the sera of some mice. However, surprisingly the presence of serum Abs did not always correlate with the induced resistance; a considerable number of mice vaccinated with PS4-BMDC produced very little or no detectable IgG or IgM Ab in their sera, but were, nevertheless, fully resistant to the Pn challenge. Furthermore, sera derived from immunized and resistant mice with no detectable anti-PS4 serum Abs failed to passively confer resistance to naïve non-immunized mice (data not shown). We observed a similar phenomenon previously, when we found that about half of the mice vaccinated with PS conjugated to an HSP60 peptide made very little to no detectible Ab in their sera, despite the fact that the conjugate vaccine had endowed these mice with resistance to lethal Pn challenge [25]. These observations raise the possibility that the protective Abs might reside in the peritoneum despite their absence from the serum. Indeed, the lack of bystander protection across serotypes (Fig. 1) indicates that the effector mechanism of resistance does discriminate between PS serotypes.

Along with specific anti-PS Ab in some of the vaccinated mice, we detected specific activation of lymphocytes in the popliteal LN draining the site of injection. We found that vaccination with LPS-stimulated PS-pulsed BMDC induced a marked increase in the size of the draining popliteal LN and in the number of isolated LN cells of about 8–10-fold compared to a naïve popliteal LN. Interestingly, immunization with LPS-stimulated unpulsed BMDC induced the same degree of LN enlargement and cell number. This suggests that the LPS-stimulated BMDC induced a local inflammatory response in the draining LN leading to a polyclonal cell proliferation. However, immunization with PS-pulsed BMDC induced a PS-specific proliferation of lymphocytes detected *ex vivo* four days later; this specific proliferative response could not be induced in mice injected with un-pulsed BMDC; immunization with PS4 induced proliferation specific to PS4 and not to PS3 and *vice versa*; this PS-specific proliferation was limited to day 4. Interestingly, resistance to lethal Pn challenge IP could be detected only from day 5 post-vaccination, a day after the LN-cell proliferation had faded. We found that the proliferation of both CD3+ T cells and CD19+ B cells was associated with IFNγ secretion in a PS-specific manner. Similarly it was shown by others that specific CD4+ T cells could respond to meningococcal PS [26]. Thus, we might hypothesize that the antigen-specific response detected in the draining LN on day 4 probably plays a role in the generation of antigen-specific resistance to challenge detected in response to IP challenge only after day 4.

Despite the induction of PS-specific CD3+ T cell proliferation, deficiency of either αβT or γδT cells did not affect the induction of resistance, whereas complete deficiency of B cells did. It is not clear whether double deficiency of both αβT and γδT cells could affect the induction of resistance. However, it would be premature to conclude that the T cells responding specifically in the popliteal LN in wild-type mice (Fig. 5) play no role in the induction of resistance; T-cell deficient mice develop alternative and compensatory mechanisms during development [27] and their immune systems are not simply the same as the wild-type immune system but only lacking T cells. Therefore, the roles of the T cell responses we observed in wild-type mice still need to be resolved. Likewise, we need to investigate which, if any cells can compensate for the congenital deficiency of T cells in the response of the knockout mice to PS-pulsed BMDC. Since both αβT and γδT cells can be the source of IFNγ [16], [28], it needs to be determined whether IFNγ is secreted in PS4-BMDC immunized TCRβ or TCRδ deficient mice and not in the double deficient mice.

Protection against the Pn challenge has been associated with rapid elimination of the bacteria by immune cells [1]. Death from Gram-positive bacteremia is accompanied by rapid growth of the bacteria [29] and the release of bacterial components [30]. Death apparently results from the overwhelming toxic-shock response of the immune system to bacterial components [30]. In our model, Pn-induced bacteremia causes death within 24 hours. Naïve mice challenged with Pn had over 10 million bacteria (CFU) per ml in their blood after 0.5 and 2 hours post-challenge before they died. In contrast, mice immunized with PS4-BMDC had not a single bacterium detectable in the blood 0.5–18 hours after Pn challenge. Surprisingly, we found that the injected Pn4 bacteria transiently replicated in the peritoneum of PS4-BMDC immunized mice and were eradicated only after about 18 hours. In the unpulsed-BMDC immunized mice, in contrast, the bacteria continued to replicate untill the mice died (Fig. 8). We hypothesize that bacteria may be trapped in leukocytes with delayed killing. Further experiments will need to be performed to determine if this hypothesis is correct.

In summary, the results obtained here indicate that PS-pulsed BMDC can induce specific adaptive responses that were not elicited by a single injection of the PS antigen itself or by a lysate of the pulsed BMDC; the draining LN seems to be the site in which the primary response develops specifically on day 4 after regional injection of the pulsed BMDC; both T cells and B cells take part in the antigen-specific LN response, but T cells are not essential in mice that lack T cells congenitally; resistance to otherwise lethal bacterial challenge in the peritoneum involves antigen-specific trapping of the bacteria, but allows them to proliferate *in situ* before being killed; and some mice resist bacterial challenge without producing IgG antibody detectable in the blood. This comprehensive view of the development of antigen-specific immunity has uncovered novel observations for focused future studies.

## Materials and Methods

### Ethics statement

All animal experiments were conducted at the Weizmann Institute of Science and approved by the Weizmann Institutional Animal Care and Use Committee (IACUC) (Permit Number: 04630909-3) according to the Israel law and the National Research Council guide (Guide for the Care and Use of Laboratory Animals 2010).

### Mice

Female C57BL/6 mice were purchased from Harlan Olac (Bicester, UK). Female B6.129P2-Tcrb^tm1Mom^/J, B6.129P2-Tcrd^tm1Mom^/J were purchased from the Jackson Laboratory (Bar Harbor, ME). CCR7 deficient mice (C57BL/6 background) [31] were kindly provided by Guy Shakhar (The Weizmann institute of Science). CD80/86 [32], MHCII [33], CD40 [34] and beta2M [35] deficient mice. All the mice were used at the age of 8–10 wk.

### Reagents

Lyophilized pathogenic Pn type 3 and type 4 bacteria and PS4 and PS3 (∼100 KD) were purchased from ATCC (Manassas, VA); LPS Salmonella Minnesota was purchased from Sigma-Aldrich (Rehovot, Israel); Alexa fluor 633, CFSE (Molecular probes, Invitrogen; Carlsbad, CA) Glutaraldehide (GH), and Paraformaldehyde (PFA) were purchased from Merck (Darmstadt, Germany) and Methyl-^3^H-Thymidin from Amersham Biosciences (Piscataway, NJ).

### Preparation of BMDC

BMDC were prepared as described earlier [12]. Briefly, mice were sacrificed and BM was extracted from femura and tibiae by flushing the shaft with PBS. Red blood cells were lysed using red blood lysis buffer (Sigma-Aldrich), and the remaining cells were seeded on non-tissue culture Petri plates at a density of 4×10^6^ cells per plate in medium (RPMI-1640, 10% FCS, 5×10^−5^ M 2-mercapto ethanol, penicillin/streptomycin) containing mouse recombinant GM-CSF 40 ng/ml (ProsPec; Rehovot, Israel). The culture medium was replenished every 3 days and the loosely adherent DC were collected after 8–10 days and used for further studies. To induce DC maturation, day 8 cultures were treated with LPS (1 µg/ml) for 24 hours.

### Purification of naïve splenic B and T cells

Spleens from naïve mice were isolated and meshed. Spleen cell suspensions were depleted of red blood cells by treatment with red blood lysis buffer (Sigma-Aldrich). B cells were then purified by negative selection with a B cell isolation kit containing biotin-conjugated mAbs to CD43, CD4, and Ter-119 (Miltenyi Biotec; Auburn, CA). T cells were purified by positive selection of CD3^+^ cells using a T cell isolation kit (Miltenyi Biotec).

### Cytokine determination in culture supernatants

The presence of cytokines in culture supernatants was determined by ELISA for IFNγ (Endogen Inc.; Boston, Mass) and IL-17 (BD Pharmingen, San Diego, CA) following the manufacturer's instructions using Maxisorp 96-well plates. Standard curves were established using mouse recombinant cytokines.

### PS4 labeling with Alexa fluor 633

The preparation of labeled PS4 was performed in collaboration with Mati Fridkin (Weizmann Institute of Science): PS4 was dissolved in DDW (10 mg/ml), and 15 mg (0.1 mmol) of 1,3-diaminopropane di-hydrochloride dissolved in DDW was added. N-ethyl-N′-(3-dimethylaminopropyl)-carbodiimide (EDC⋅HCL) (19 mg; 0.1 mmol) (Sigma-Aldrich) was then added, and the reaction mixture was kept for 4 hours at room temperature. Then another portion of EDC⋅HCL (9.5 mg; 0.05 mmol) was added. After 10 hours at room temperature, the clear homogenous reaction mixture was extensively dialyzed against 0.1N acetic acid at 4°C to remove low-molecular weight molecules. The pH was adjusted to 7.5 with 0.1N KHCO_3_ solution followed by the addition of Alexa fluor 633 (19 mg; ∼0.05 mmol). After standing overnight at room temperature, the product solution was dialyzed extensively against DDW at 4°C and then against 0.05N acetic acid. The solution was divided into aliquots and kept at 4°C with 0.01M sodium azid.

### Flow cytometric analysis and immunocytochemistry

The presence of PS4 on the BMDC surface was observed using flow cytometry. BMDC (1×10^6^ per tube) were pulsed with the Alexafluor 633 labeled-PS4 at 4°C or 37°C as indicated in the text. The cells were washed with PBS/1% BSA and then incubated for 15 min with the anti-FcγRIII/II 2.4G2 Ab (BD Pharmingen, San Diego, CA) to block nonspecific binding. After washing, the cells were stained with specific surface marker Abs: anti-mouse CD1d-PE, I-AE-PE (eBioscience; San Diego, CA), CD11c-APC (Biolegends; San Diego, CA) or H-2K^b^-FITC (CALTAG Laboratories) for an additional 15 minutes on ice. The cells were then washed and transferred to 10 mm glass cover-slips (Deckglaser, Germany) in 24-well plates (2×10^5^/well) for 30 minutes at 37°C to allow adherence. The cells then were fixed with 4% PFA for 15 minutes at room temperature. The coverslip were put upside down on slides with elvanol. The cells were also analyzed by flow cytometry analysis using the FACSort machine and the CellQuest software (BD Biosciences).

### Vaccination with PS-pulsed BMDC, with un-pulsed BMDC or with soluble PS

BMDC were grown as described above. Part of the cells was stimulated with LPS 1 µg/ml O/N to induce maturation. LPS-stimulated and non-stimulated BMDC were collected into conical tubes and incubated with PS 100 µg/ml (per 5×10^6^ cells) for 1 hour at 37°C. Then the cells were harvested and extensively washed with ice–cold PBS to remove unbound PS and were injected IP (200 µL per mouse) or IFP (25 µL per mouse). Control LPS-stimulated unpulsed BMDC or soluble PS (100 µg/ml) and LPS (1 µg/ml) were injected as well.

### Pn bacteria

Lyophilized Pn type 3 or type 4 bacteria and PS antigen were obtained from the ATCC. Pn bacteria were reconstituted and sub-cultured on sheep's blood agar (Hy Laboratories, Rehovot, Israel) and the colonies were re-suspended in brain-heart infusion (BHI) broth (Hy Laboratories). After 6 h of growth, the bacterial cultures were aliquoted and stored at −80°C in medium with 25% glycerol. For pneumococcal challenge, a frozen aliquot of Pn bacteria was thawed in BHI broth and was grown for 6 h at 37°C, and then transferred to ice until injection. Bacterial growth was estimated by the level of turbidity using an OD reader at 545 nm. The actual dose of viable bacteria injected in each challenge was determined by plating dilutions of the bacteria on sheep's blood agar for 24 h at 37°C and counting the number of CFU; one CFU was considered to represent one bacterium sown on the plate at a limiting dilution. Pneumococcal virulence was maintained by periodic passage in mice: mice were injected with 100 Pn CFU, and 20 h later the spleens were harvested, passed through a wire mesh, and seeded on sheep's blood agar. The bacteria were then prepared as indicated above. To determine the minimal lethal dose, naive C57BL/6 mice (3 mice per group) were injected IP with 200 µl of serially diluted bacteria, and survival was determined daily for 2 wk. All naive mice challenged with two or more CFU died within one day of challenge. The minimal lethal dose that killed half of the challenged mice (LD_50_) was determined to be one bacterium per mouse.

### ELISA serology

Vaccinated mice were bled two days before bacterial challenge and serum samples were tested in ELISA; 96-well maxisorp plates (Nunc; Rochester, NY) were coated with the PS antigen (10 µg/ml) O/N at 4°C. The plates were washed and blocked with 100 µL of BSA 1% for 1 hour R/T. Then, dilutions of the serum samples in PBS/1% BSA (1∶100) were added for 1.5 hours R/T, then AP-conjugated anti-mouse IgG (Jackson Laboratories), IgM, IgA or IgE (Southernbiotech Lab; Birmingham, Alabama) for 1 hour R/T and finally phosphate substrate. The yellowish color was read at 405 nm.

### Proliferation test of draining LN lymphocytes

Labeled-thymidin proliferation assay: Draining LN, non-draining LN and spleens were isolated and meshed. The cells were incubated in quadruplicate in round-bottom 96-well plates (5×10^5^ cells per well) at 37°C in 5% CO_2_ in 200 µl of RPMI supplemented with 1% syngeneic naïve mouse serum, 5×10^−5^ M beta-ME, L-glutamate, sodium pyruvate, non-essential amino acids, and penicillin/streptomycin. PS4 or PS3 were added (50 µg/ml) to the culture. After 85 h, the cells were pulsed with 1 µCi of [^3^H]thymidine for 12 h, and [^3^H]thymidine incorporation was measured using a 96-well plate beta counter. The mean cpm ± SD were calculated for each quadruplicate.

CFSE-based proliferation assay: Draining LN, non-draining LN and spleens were isolated and meshed. The cells were labeled with CFSE (C-1157; Invitrogen) and cultured together with PS4 50 µg/ml in medium supplemented with 1% syngeneic naïve mouse serum in 96-well plates. After 96 hours, the cells were collected and stained for anti- mouse CD3-biotin (eBioscience)∶cy5-SA (Jackson Lab) or anti- mouse CD19-APC (eBioscience). The cells were then washed and examined by flow cytometry.

### Examination of peritoneal lavage and blood samples of challenged mice by flow cytometry, and CFU counting assay

Vaccinated mice were challenged with Pn4 bacteria. Before challenge (time 0) and different time points after challenge, blood samples of vaccinated mice were collected into heparin. Blood and peritoneal lavage samples were tested for living bacteria by CFU counting assay; the samples were diluted or were put as is on blood agar plates. Twenty-four hours later, CFU were count, and the total CFU number was calculated.

### Statistics

The InStat 2.01 program (GraphPad) was used for statistical analysis. Statistical analysis was performed using the two-sided Welch T test, Wilcoxson non-parametric test or Fisher's exact Chi test. Differences were considered statistically significant at p<0.05.

## References

[1] AlonsoDeVelasco E, Verheul AF, Verhoef J, Snippe H (1995). Streptococcus pneumoniae: virulence factors, pathogenesis, and vaccines.. Microbiol Rev.

[2] Calbo E, Garau J (2011). Factors affecting the development of systemic inflammatory response syndrome in pneumococcal infections.. Curr Opin Infect Dis.

[3] Hilleman MR, McLean AA, Vella PP, Weibel RE, Woodhour AF (1978). Polyvalent pneumococcal polysaccharide vaccines.. Bull World Health Organ.

[4] Smit P, Oberholzer D, Hayden-Smith S, Koornhof HJ, Hilleman MR (1977). Protective efficacy of pneumococcal polysaccharide vaccines.. JAMA.

[5] French N, Nakiyingi J, Carpenter LM, Lugada E, Watera C (2000). 23-valent pneumococcal polysaccharide vaccine in HIV-1-infected Ugandan adults: double-blind, randomised and placebo controlled trial.. Lancet.

[6] Douglas RM, Paton JC, Duncan SJ, Hansman DJ (1983). Antibody response to pneumococcal vaccination in children younger than five years of age.. J Infect Dis.

[7] Selman S, Hayes D, Perin LA, Hayes WS (2000). Pneumococcal conjugate vaccine for young children.. Manag Care 9: 49–52, 54, 56–47 passim.

[8] Nuorti JP, Whitney CG (2010). Prevention of pneumococcal disease among infants and children – use of 13-valent pneumococcal conjugate vaccine and 23-valent pneumococcal polysaccharide vaccine – recommendations of the Advisory Committee on Immunization Practices (ACIP).. MMWR Recomm Rep.

[9] Mosser DM, Edwards JP (2008). Exploring the full spectrum of macrophage activation.. Nat Rev Immunol.

[10] Steinman RM (1991). The dendritic cell system and its role in immunogenicity.. Annu Rev Immunol.

[11] Geissmann F, Manz MG, Jung S, Sieweke MH, Merad M (2010). Development of monocytes, macrophages, and dendritic cells.. Science.

[12] Cohen N, Stolarsky-Bennun M, Amir-Kroll H, Margalit R, Nussbaum G (2008). Pneumococcal capsular polysaccharide is immunogenic when present on the surface of macrophages and dendritic cells: TLR4 signaling induced by a conjugate vaccine or by lipopolysaccharide is conducive.. J Immunol.

[13] Jiang SM, Wang L, Reeves PR (2001). Molecular characterization of Streptococcus pneumoniae type 4, 6B, 8, and 18C capsular polysaccharide gene clusters.. Infect Immun.

[14] Garcia E, Lopez R (1997). Molecular biology of the capsular genes of Streptococcus pneumoniae.. FEMS Microbiol Lett.

[15] Saeki H, Moore AM, Brown MJ, Hwang ST (1999). Cutting edge: secondary lymphoid-tissue chemokine (SLC) and CC chemokine receptor 7 (CCR7) participate in the emigration pathway of mature dendritic cells from the skin to regional lymph nodes.. J Immunol.

[16] Jensen KD, Su X, Shin S, Li L, Youssef S (2008). Thymic selection determines gammadelta T cell effector fate: antigen-naive cells make interleukin-17 and antigen-experienced cells make interferon gamma.. Immunity.

[17] Vabulas RM, Ahmad-Nejad P, da Costa C, Miethke T, Kirschning CJ (2001). Endocytosed HSP60s use toll-like receptor 2 (TLR2) and TLR4 to activate the toll/interleukin-1 receptor signaling pathway in innate immune cells.. J Biol Chem.

[18] Cobb BA, Wang Q, Tzianabos AO, Kasper DL (2004). Polysaccharide processing and presentation by the MHCII pathway.. Cell.

[19] Meltzer U, Goldblatt D (2006). Pneumococcal polysaccharides interact with human dendritic cells.. Infect Immun.

[20] Stephen TL, Fabri M, Groneck L, Rohn TA, Hafke H (2007). Transport of Streptococcus pneumoniae capsular polysaccharide in MHC Class II tubules.. PLoS Pathog.

[21] Lai Z, Schreiber JR (2009). Antigen processing of glycoconjugate vaccines; the polysaccharide portion of the pneumococcal CRM(197) conjugate vaccine co-localizes with MHC II on the antigen processing cell surface.. Vaccine.

[22] Brown EJ, Hosea SW, Frank MM (1983). The role of antibody and complement in the reticuloendothelial clearance of pneumococci from the bloodstream.. Rev Infect Dis.

[23] Schiffman G (1983). Pneumococcal vaccine: a tool for the evaluation of the B-cell function of the immune system.. Proc Soc Exp Biol Med.

[24] Watson DA, Musher DM, Verhoef J (1995). Pneumococcal virulence factors and host immune responses to them.. Eur J Clin Microbiol Infect Dis.

[25] Amir-Kroll H, Nussbaum G, Cohen IR (2003). Proteins and their derived peptides as carriers in a conjugate vaccine for Streptococcus pneumoniae: self-heat shock protein 60 and tetanus toxoid.. J Immunol.

[26] Muthukkumar S, Stein KE (2004). Immunization with meningococcal polysaccharide-tetanus toxoid conjugate induces polysaccharide-reactive T cells in mice.. Vaccine.

[27] Yanez DM, Batchelder J, van der Heyde HC, Manning DD, Weidanz WP (1999). Gamma delta T-cell function in pathogenesis of cerebral malaria in mice infected with Plasmodium berghei ANKA.. Infect Immun.

[28] Szabo SJ, Sullivan BM, Stemmann C, Satoskar AR, Sleckman BP (2002). Distinct effects of T-bet in TH1 lineage commitment and IFN-gamma production in CD4 and CD8 T cells.. Science.

[29] Dallaire F, Ouellet N, Bergeron Y, Turmel V, Gauthier MC (2001). Microbiological and inflammatory factors associated with the development of pneumococcal pneumonia.. J Infect Dis.

[30] Tuomanen EI (1997). The biology of pneumococcal infection.. Pediatr Res.

[31] Forster R, Schubel A, Breitfeld D, Kremmer E, Renner-Muller I (1999). CCR7 coordinates the primary immune response by establishing functional microenvironments in secondary lymphoid organs.. Cell.

[32] Schweitzer AN, Borriello F, Wong RC, Abbas AK, Sharpe AH (1997). Role of costimulators in T cell differentiation: studies using antigen-presenting cells lacking expression of CD80 or CD86.. J Immunol.

[33] Grusby MJ, Johnson RS, Papaioannou VE, Glimcher LH (1991). Depletion of CD4+ T cells in major histocompatibility complex class II-deficient mice.. Science.

[34] Kawabe T, Naka T, Yoshida K, Tanaka T, Fujiwara H (1994). The immune responses in CD40-deficient mice: impaired immunoglobulin class switching and germinal center formation.. Immunity.

[35] Koller BH, Marrack P, Kappler JW, Smithies O (1990). Normal development of mice deficient in beta 2M, MHC class I proteins, and CD8+ T cells.. Science.

